# Adaptation of Some Quinoa Genotypes (*Chenopodium quinoa* Willd.), Grown in a Saharan Climate in Algeria

**DOI:** 10.3390/life12111854

**Published:** 2022-11-11

**Authors:** Kelthoum Maamri, Ouiza Djerroudi Zidane, Ahmed Chaabena, Gabriele Fiene, Didier Bazile

**Affiliations:** 1 Research Laboratory on Phoeniciculture, Faculty Science of Nature and Life, Kasdi Merbah Ouargla University, Ouargla 30000, Algeria; 2 Saharan Bio-Resources Laboratory, Safeguarding and Valorization, Kasdi Merbah Ouargla University, Ouargla 30000, Algeria; 3 CIRAD, UMR SENS, F-34398 Montpellier, France; 4 SENS, Univ Montpellier, CIRAD, F-34398 Montpellier, France

**Keywords:** adaptation, *Chenopodium quinoa* Willd., genotypes, salinity, Sahara, Algeria

## Abstract

Agriculture in southern Algeria faces several challenges that hinder its development, including drought, high temperatures and the excessive salinity of soil and groundwater. The introduction of crops resistant to these factors is one of the solutions chosen to address these abiotic constraints. This research aimed to evaluate the behavior of quinoa *(Chenopodium Quinoa* Willd.) grown in the Ouargla region of southeastern Algeria. Five varieties of quinoa (*Santa maria, Giza1, Amarilla Sacaca, Blanca de Junin* and *Kancolla)* were tested at two sites that differed in terms of soil salinity (9.95 mS/cm and 0.85 mS/cm) during 2019 and 2020. A complete random block experimental design with four repetitions was used for the agronomic tests. Our results clearly show that higher grain yields were obtained at the high salinity site (site 1) compared to the low salinity site (site 2). However, plant height, grain yield per plant and harvest index differed between varieties and sites. In contrast, stem diameter was not greatly affected by salinity. The varieties that seem to be best adapted to the growing conditions of the Ouargla region are, in descending order: *Santa Maria, Giza1, Amarilla Sacaca* and *Blanca de Junin*. When testing quinoa in new environments, it is critical to adapt the cropping cycle of varieties to avoid very high temperatures. The choice to switch to winter cultivation instead of spring cultivation can be an essential criterion for success. The biogeographical approach conducted in this research opens up new perspectives for the adaptation and cultivation of quinoa outside its region of origin to satisfy the food security of the people of North Africa.

## 1. Introduction

*Chenopodium quinoa* Willd., a plant native to the Andean highlands, was first domesticated around Lake Titicaca, which lies at an altitude of 3800 m along the Peruvian-Bolivian border [1]. The domestication of quinoa began about 7000 years ago [2], and the process is considered to be ongoing today as the crop continues to be adapted to new environments. For centuries, quinoa was a staple food for the people of the Andes [3,4,5]. Today, its use is mainly based on human consumption of the grains, such as with cereals.

Quinoa is an optional halophyte plant [6]. A dicotyledonous herbaceous belonging to the Amaranthaceae, the fruit is a tiny achene whose seed color varies between white, yellow, purple and black [7]. Quinoa is one of the most nutritious food crops currently known in the world. The seeds contain high-quality proteins, as they possess all nine essential amino acids, including lysine, methionine and threonine, which are rare and often a limiting factor in cereals and legumes [8,9].

Worldwide, there are over 6000 accessions of quinoa grown by farmers [10]. The genetic diversity of the quinoa species can be classified into five major ecotypes [11] (highlands, inter-Andean valley, *salares* (salt lakes), *yungas* (subtropical forests) and coastal lowlands) according to their adaptation to the specific agro-ecological conditions of the main production areas [2,12,13]. The high genetic diversity of the species offers opportunities to take advantage of its hardiness and promote its wide adaptation [14]. The needs of the crop vary considerably depending on the local variety or cultivar [15].

The hardiness of the species allows quinoa to thrive in a wide range of climatic conditions, including desert-like, hot, dry, cold and temperate and rainy, and hot with high humidity [1,16,17,18]. Several scientific studies have confirmed that thanks to physiological mechanisms, quinoa can tolerate very dry conditions and drought [19].

The ideal average temperature for quinoa development and growth is around 15 °C to 20 °C, but some varieties can also withstand extreme temperatures of −8 °C to +38 °C [20].

Periods of temperature sensitivity have been recorded, mainly when seed germination occurs at cold temperatures (frost) and when flowering occurs at high temperatures [21,22]. There are varieties adapted to short days or long days, and there are others insensitive to photoperiod [23,24]. Depending on the photoperiod sensitivity of each variety, the duration of growth can be modified according to the length of days and temperatures [25,26]. Photoperiod sensitivity is a key factor in the adaptation of this crop to new latitudes [24], and should be considered along with the analysis of the distribution of daily temperatures during the crop development cycle.

Quinoa has exceptional nutritional properties, with a high protein content compared to cereals, combined with a good balance and sufficient content of all essential amino acids [27,28,29,30]. Quinoa thus represents an opportunity for farmers exposed to an increasingly drier climate [31]. It is also one solution for the rehabilitation of salt-affected land, as quinoa is considered one of the most promising food crops for sustainable agriculture in regions affected by soil and water salinization [32]. This is why its status as a facultative halophyte makes it an alternative cash crop for land and water unsuitable for conventional crops in arid and semi-arid regions [33,34]. Quinoa has the ability to grow and complete its life cycle under high salinity levels that are almost similar to those found in seawater [35,36,37].

Quinoa is a viable alternative in areas limited by climate change and soil salinization. These constraints affect the conditions under which crops can grow and influence the nutritional quality of the grains. Soil and water salinity is ubiquitous, with about one billion hectares affected worldwide in 2021 [38]. Against this backdrop, quinoa appears to be a hardy crop with interesting agronomic and physiological traits. It can grow under different stress conditions such as soil salinity and acidity, and can under certain conditions tolerate episodes of drought and frost [1,39]. This ability of quinoa to grow under extreme stress conditions has encouraged researchers to take it out of the Andes and attempt to adapt it to other parts of the world [20]. Today, various studies are investigating its adaptation in Europe [40], but also and above all in marginal arid and semi-arid zones [41].

Given that the Earth’s population will reach nine billion within the next few decades, global food security is becoming an increasingly urgent concern. Today, 870 million people already suffer from hunger in underdeveloped countries [7], and two billion people are estimated to be undernourished [42].

Faced with these challenges, the hardiness of quinoa, linked to the species’ very high genetic diversity, and it’s incredible nutritional richness mean that it is increasingly appreciated by producers and researchers. Today the plant is cultivated or under testing in over 125 countries [43] and its cultivation continues to develop rapidly [41,44,45,46].

The spatial and temporal expansion of quinoa around the world went through several stages [47] over time. During phase 1 (before the 1900s), quinoa was limited to the Andean countries (Colombia, Ecuador, Peru, Bolivia, Argentina and Chile). It was considered a local food crop and a staple food for Andean populations [46,48,49]. In phase 2 (between 1901 and 1969), quinoa was imported into Africa as an experiment. The first known trial outside the Andes took place in 1935 in Kenya, and other trials on quinoa’s response to nutrient deficiencies and tolerance to abiotic stresses (salinity and temperature) were conducted between 1950 and 1968 [47,50]. In phase 3 (between 1970 and 1989), quinoa was introduced into northern continents, North America (Colorado (USA) [50,51]), Europe (England, Denmark and the Netherlands [1,50,52]), and Asia (India and China [46,50,53]). During this period, quinoa was also tested in Brazil and Cuba [20,50]. At the end of the 1980s, quinoa was present in 11 countries outside the Andes. During phase 4 (between 1990 and 2012), quinoa spread to 30 new countries, propelled by the project “American and European Test of Quinoa” between 1996 and 1998 [1,47], which gave birth to the first variety (Atlas) and cultivars (Carmen) in Europe [1,46]. In 2012, quinoa appeared in a few countries in the Mediterranean region [47,50]. In phase 5 (between 2013 and 2018), following the declaration of the International Year of Quinoa in 2013, it was tested in 76 countries: 31 in Africa, 24 in Asia, and 15 in Europe [46]. An FAO regional project entitled “Technical Assistance for Strengthening the Food System Associated with Quinoa”, was also launched in 2013–2015, implementing the distribution of quinoa accessions among national research institutions in eight countries of North Africa and the Middle East (Algeria, Egypt, Iraq, Iran, Lebanon, Mauritania, Sudan and Yemen) [41] to evaluate these genotypes under semi-arid and arid conditions.

**Figure 1:**
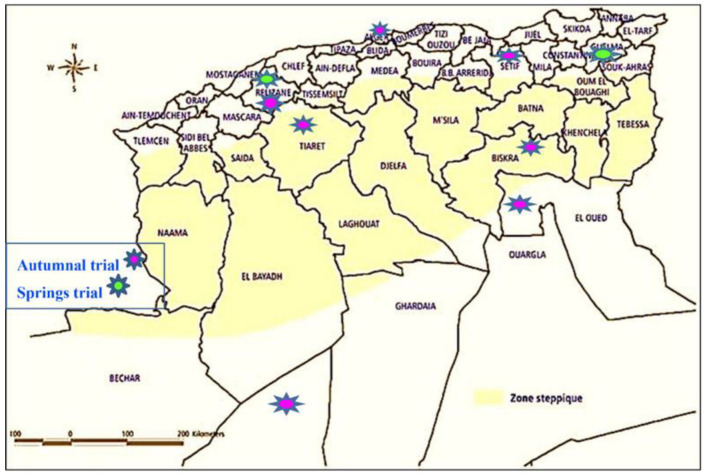
Location of the first quinoa cultivation trials in Algeria. *Own elaboration adapted from a personal communication from the Technical Institute for the Development of Saharan Agriculture (ITDAS)*.

Algeria is one of the countries that has benefited from the expansion of quinoa thanks to the scientific and technical expertise provided by the FAO to assess the behavior of this crop when it was first introduced into the country in 2013–2014. During this first experiment, eight trial sites were chosen to represent the different agro-ecological regions of the country. These were Baïnem (Algiers), Setif, Tiaret, Relizane, Guelma, Biskra, El Oued and Adrar. International cooperation under the aegis of FAO made it possible to evaluate 16 quinoa genotypes (Q21, Q12, Q29, Q18, Q26, Q22, Q27, Giza1, Giza2, *Sajama, Santamaria, Amarilla Marangani, Amarille Sacaca, Blanca de Junin, Kancolla* and *Salcedo Inea*) under arid and semi-arid conditions in order to characterize the phenological development of plants and determine the yield components according to the selected varieties and sites. The first trials were carried out in the autumn of 2014 at seven sites, namely Baïnem (Algiers), Setif, Tiaret, Biskra, El Oued, Adrar and Relizane, and trials were conducted at the other two sites, Guelma and Relizane, the following spring of 2015 (Figure 1). The yield of these trials ranged from 0 to 2.62 t/ha. Despite the various experimental trials carried out on these experimental stations, some with good agronomic results, the cultivation of quinoa has remained at an elementary stage and has not yet met the conditions to be generalized, or better known, across Algeria. New research is currently being carried out on the morphological characterization of certain varieties of quinoa, as well as the effect of saline stress on the physiological performance of these plants. This article presents the first results of the most recent research conducted in Algeria in the arid region of Ouargla. The primary objective of the introduction of quinoa in Algeria is to find alternative species in order to continue to exploit marginal lands affected by salinity, drought and very high temperatures. The aim is to determine whether quinoa is hardy enough to cope with the current and future challenges of the Saharan agrosystem and withstand desert conditions that continue to deteriorate.

To gain a better mastery of quinoa cultivation techniques in Algeria, multiple studies in the different agro-ecological regions of the country are required. The present study was conducted during 2019 and 2020 to assess the behavior of quinoa and its growth under Saharan conditions.

## 2. Materials and Methods

### 2.1. General Presentation of Agriculture in Algeria

Algeria is the largest country in Africa—since the splitting of Sudan—with an area of 2,381,741 km^2^. Due to its vast surface area and distinctive geographical position, extending from the shores of the Mediterranean to the Sahara, the country has a wide range of climates. The northern part, which extends from the Mediterranean coast and includes the Tell Atlas, has a Mediterranean climate, while the rest of the country has a predominantly desert (Saharan) climate (Table 1). Between these two major climatic types, there are many transitional climates in the space between the Tell Atlas and the Saharan Atlas mountain chains, including a semi-arid climate, which corresponds to a Mediterranean climate with a persistent drought over a large part of the year [54]. As a whole, and despite its northern facade and 1200 km long Mediterranean coastline, Algeria is much more semi-arid and arid than humid, with a dominant climate that is hot and dry for most of the year. The desert part (Sahara) covers more than 89% of the country, or about 2 million km^2^, while the utilized agricultural area (UAA) covers 8.5 million ha, representing about 19.7% of the country’s surface, of which 15% is irrigated. The ratio of hectares per capita is also the lowest in the Maghreb region; it is estimated at 0.19 ha/inhabitant, compared to 0.27 ha/inhabitant for Morocco and 0.45 ha/inhabitant for Tunisia [55].

**Figure 2:**
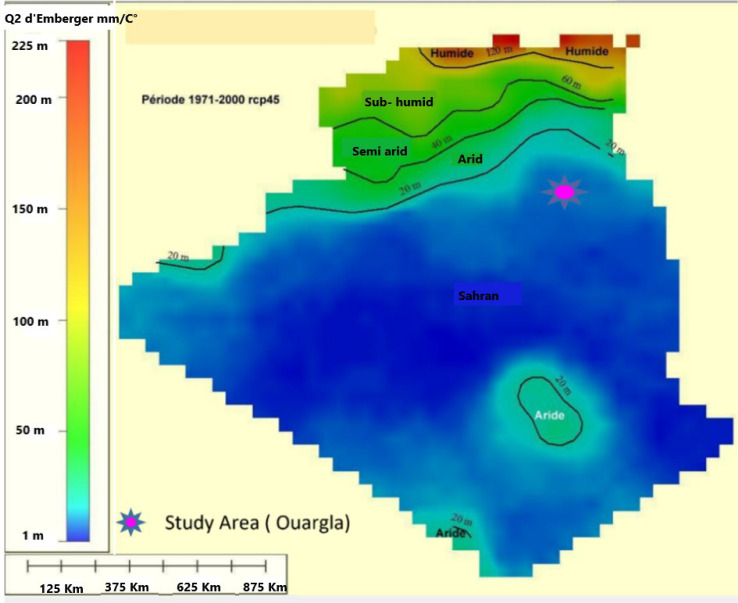
Distribution of bioclimatic zones in Algeria according to Emberger’s Q2 measurement [54]. *Own elaboration adapted from the Commissariat for the development of agriculture in the Sa-haran regions (CDARS from the study on the improvement of livestock conditions in Saharan rangelands (CDARS/Ministry of Agriculture and Rural Development, 2017)*.

In Algeria, field crops, particularly cereals, occupy more than half of the UAA and are mainly found in semi-arid areas, highlands and sub-humid areas (Figure 2). Arboriculture occupies just over 10% of the UAA and is represented by olive, date palm and other fruit trees. Vegetable crops cover about 5% of the UAA [56].

**Figure 3:**
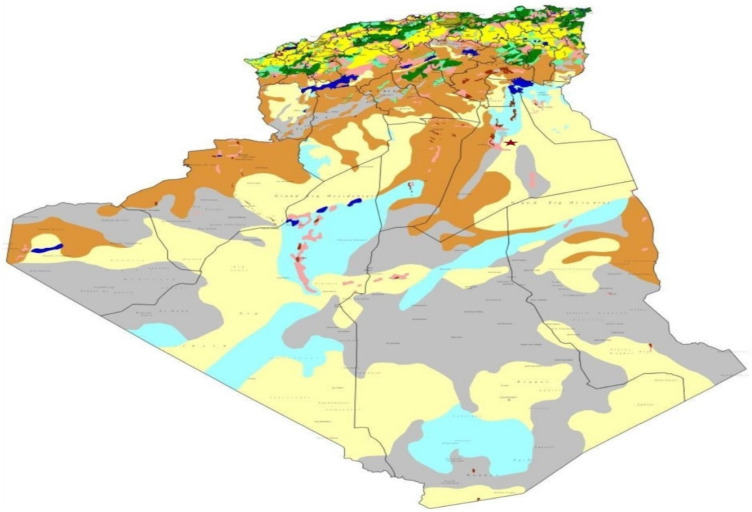

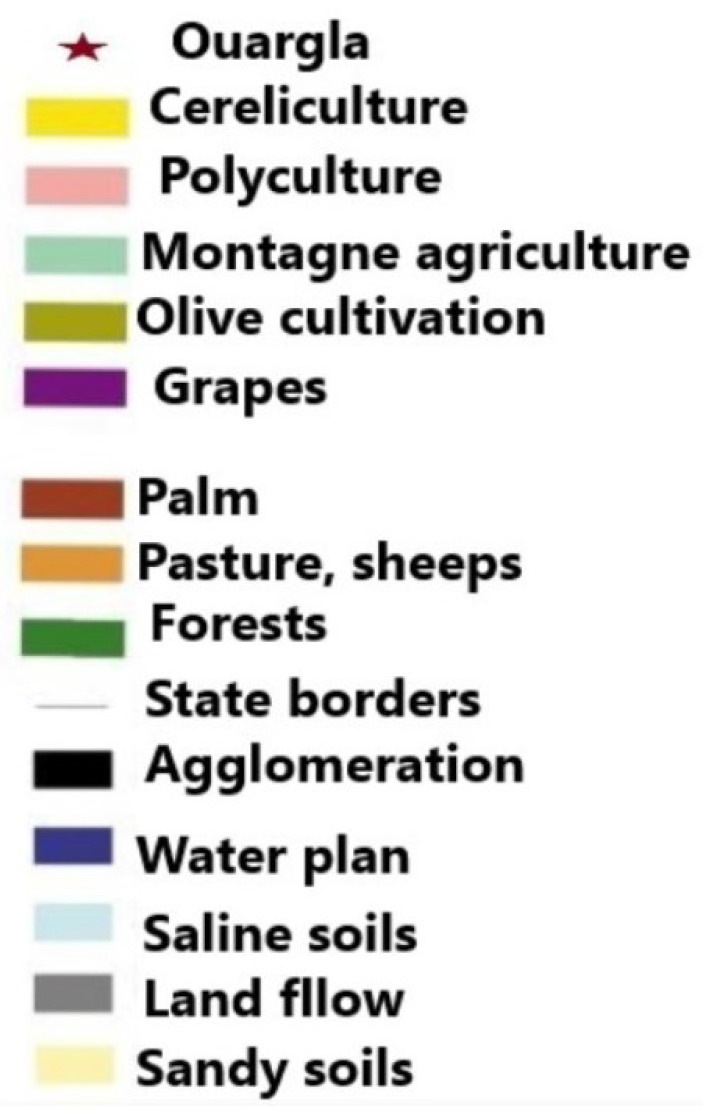
Agricultural regions in Algeria [58]. *Own production adapted from the Ministry of Agriculture and Rural Development (MADR, 2007)*.

The agriculture of the oases in the south is organized around gardens planted with date palms, associated with market gardening and fruit trees, irrigated by traditional techniques (submersion, seguia (Open-air water supply pipes for irrigation, usually made of earth) and foggaras (Underground pipe (draining gallery), to bring water from upstream to downstream, for agricultural and other needs, located in the region of Touat, Gourara and Tidikelt)). The extension of agriculture in the south outside the oases is progressing in the form of modern land development schemes created under the Law on Access to Agricultural Land Ownership of 1983 (APFA). Most are near traditional palm groves [57]. These are mainly oriented towards pivot cereal farming and date palms irrigated by drip systems (Figure 3).

### 2.2. Challenges and Constraints of Saharan Agriculture

The Algerian Sahara is a huge biogeographical entity covering 2,000,000 km². This natural area faces climatic, soil and anthropogenic challenges, with consequences for the degradation of Saharan agriculture and existing cropping systems, and consequently on the food and nutrition security of the population. The rainfall regime of the Sahara is characterized by low rainfall of about 150 mm per year north of the Sahara, but less than 50 mm in most other Saharan regions along with very high temperatures (over 40 °C), which accentuate the effects of drought [59,60]. Winds also are an aggravating factor, and they are challenging due to the transport of sand that they cause. They are relatively frequent and their speeds are important from April to July, which causes the *siroco* (or sand wind) responsible for silting phenomena with the formation and displacement of dunes [60]. All of these negative conditions make it impossible to grow crops without irrigation in the Saharan zone.

Other constraining aspects of the Saharan climate include both the very high daily thermal amplitude and the annual thermal amplitude. The very low temperatures recorded during the first three months of the year cause frosts and are a limiting factor to be taken into account for crop cycles [59]. Sahara soils are generally composed of sandy mineral substrates, devoid of organic matter, with a coarse texture, low water retention capacity, and limited depth. The Saharan waters are generally chlorinated, chlorinated-sulphated, or sulphated-chlorinated. The chlorine concentration of irrigation water is generally greater than 10 mEq/L [59]. The quality of irrigation water is most often poor because of this primary salinity of geological waters. This is further increased by poor water resource management, which is called secondary salinization [61]. The salinity of the waters is probably one of the main reasons for the low yields obtained in certain irrigated areas of Algeria. The magnesium concentration of irrigation water also is sometimes high. Finally, it appears that these waters have a high ion concentration, which gives them a high risk of salinization and generates risks of toxicity by Na and Cl ions [59].

### 2.3. Study Sites

The wilaya of Ouargla is located northeast of the northern Sahara. It covers an area of 163,233 km^2^. It is characterized by an arid climate, with an average monthly temperature of 42.8 °C in July and a minimum average of 4 °C in January. The average annual rainfall is 50 mm. The texture of the soil is usually sandy or sandy-silty.

**Figure 4:**
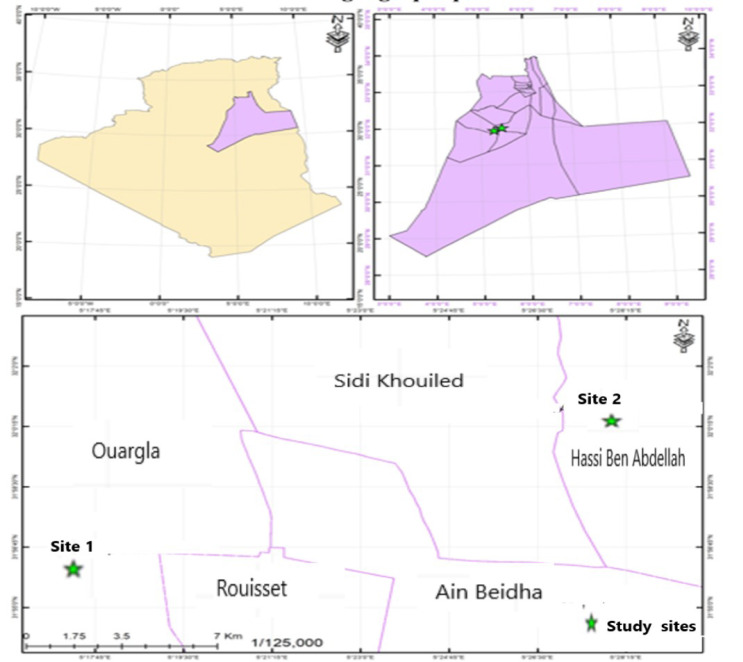
Location map of study sites.

The trials conducted for this research were conducted during the winter period of the 2019/2020 campaign to compare two different sites in the Ouargla region, both in open fields and under irrigation. The first site (1) is located in the experimental farm of the Faculty of Natural Sciences and Life of Kasdi Merbah University in the municipality of Ouargla (31°56′20.82″ N latitude, 5°17′33.71″ E longitude, altitude 246 m). The second site (2) is located at the Technical Institute for the Development of Saharan Agriculture, which is in the municipality of Hassi Ben Abdellah (ITDAS), with coordinates 32°0′25.59″ N latitude, 5°27′48.63″ E longitude, altitude 446 m (Figure 4).

Groundwater is the main source of irrigation water used by farmers in the region. The first site is irrigated by the Mio-Pliocene aquifer with an EC = 3.03 mS/cm and a pH = 7.71, while the second site is irrigated by the Intercalary continental (CI) aquifer or the Albian (EC = 2.45 mS/cm and pH = 7.87).

The soil is sandy in texture with an alkaline pH (pH = 7.89 and 7.54) for both sites, but they differ in terms of EC; the first site has highly saline soil, while the second site has low salinity.

The same experimental set-up was adopted in both sites, consisting of a randomized complete block design with four replications. Each treatment (genotype) was represented only once in each block and the distribution of treatments was randomized. The area of each elementary plot was 10 m². Each plot comprised five rows spaced 40 cm apart; the inter-plant spacing was 20 cm. Measurements were carried out on the plants in the middle row of each plot. The trials were carried out in the open field and under drip irrigation, with an organic fertilization of 40 t/ha. Sowing was done on 17 and 26 October 2019 for sites 1 and 2, respectively.

The Ouargla region is characterized by a Saharan climate, with very low rainfall, high temperatures, and high evaporation (Table 2). The annual average maximum and minimum temperatures measured in our study were 31.2 °C and 16.5 °C, respectively; the highest temperature recorded was 48.2 °C during the month of July and the lowest −1 °C in January. Rainfall is rare and irregular, and the total annual rainfall is 13.21 mm.

### 2.4. Plant Material Used

The plant material used in this study included five genotypes of quinoa (*Chenopodium quinoa* Willd.). These were *Santa Maria*, Giza1, *Amarilla Sacaca*, *Blanca de Junin* and *Kancolla*, which were provided by ITDAS and whose seeds were produced in the FAO trial plots. Table 3 shows some characteristics of the seeds used.

### 2.5. Morphological and Agronomic Measurements

The choice of indicators for characterization and monitoring of plant development was made using the book “Descriptors for Quinoa (*Chenopodium quinoa* Willd.) and its Wild Relatives” (Bioversity International, FAO, PROINPA, INIAF and IFAD). The indicators selected to be measured at harvest (physiological maturity) were: plant height (PHT), stem diameter (SD), grain yield per plant (GYP), harvest index (HI) calculated as the ratio of GYP to total shoot dry matter, and the number of days from sowing to maturity.

Considering the BBCH Method applied to quinoa, a field is at stage when 50% of the plant has the corresponding development level [62,63]. For harvesting, a growth stage above 95 was considered as physiological maturity and for plant measuring.

### 2.6. Statistical Analyses

For morphological and agronomic measurements, an analysis of variance (ANOVA) was conducted using XL-STAT software (2014), and parameter means were compared using a Tukey’s test (*p* ≤ 0.05).

## 3. Results

### 3.1. Number of Days of Genotype Growth

**Figure 5:**
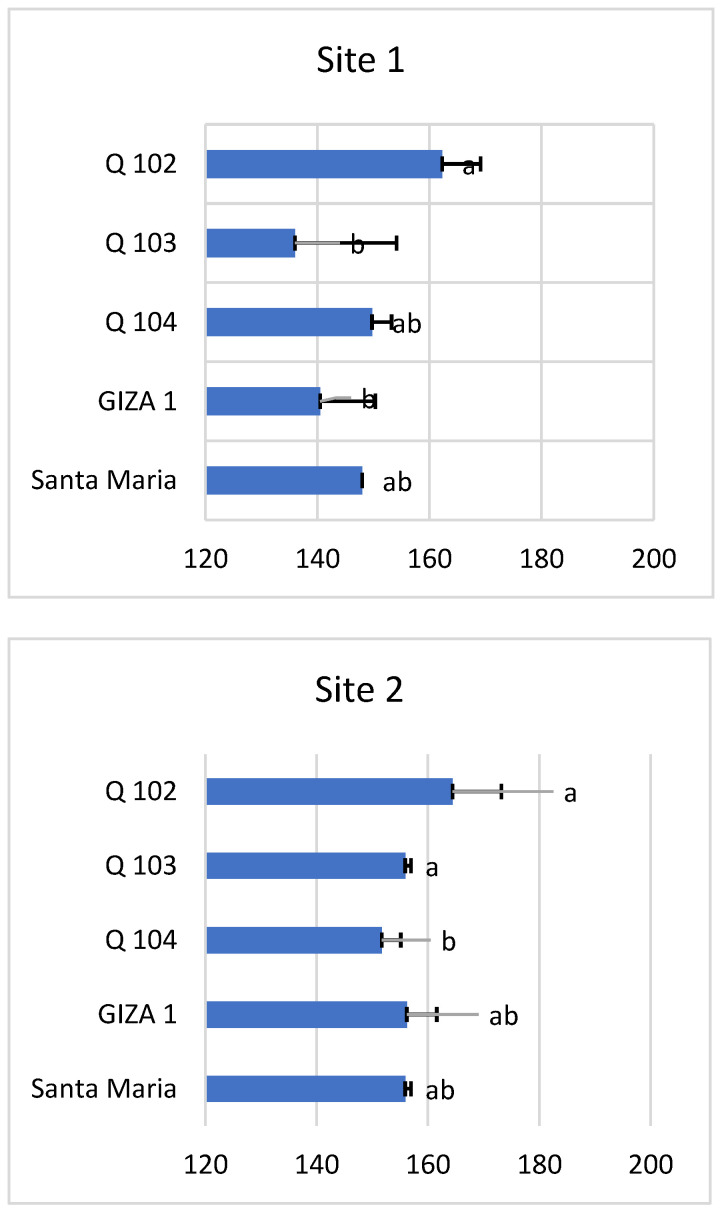
Total duration of the growth cycle of quinoa varieties at both sites. *Means with different letters showed statistical difference (p ≤ 0.02 for site 1 and p ≤ 0.03 for site 2)*.

The statistical analysis revealed a significant difference in the number of days until grain maturity between the varieties studied, and this in the two study sites (site 1 *p* < 0.02 and site 2 *p* < 0.03) (Figure 5). Genotype Q102 had the longest maturity time at both sites, with 162 and 164 days for sites 1 and 2, respectively, while the shortest maturity time was 136 days for Giza1 in site 1 and 152 days for Q104 in site 2. All of the varieties reached maturity proportionally earlier under the conditions of the first site compared to those of the second site.

This difference can be explained by the difference between the date of sowing and the altitude. Temperatures decrease at higher altitudes, which lengthens the growing cycle for the same photoperiod.

### 3.2. Plant Height

**Figure 6:**
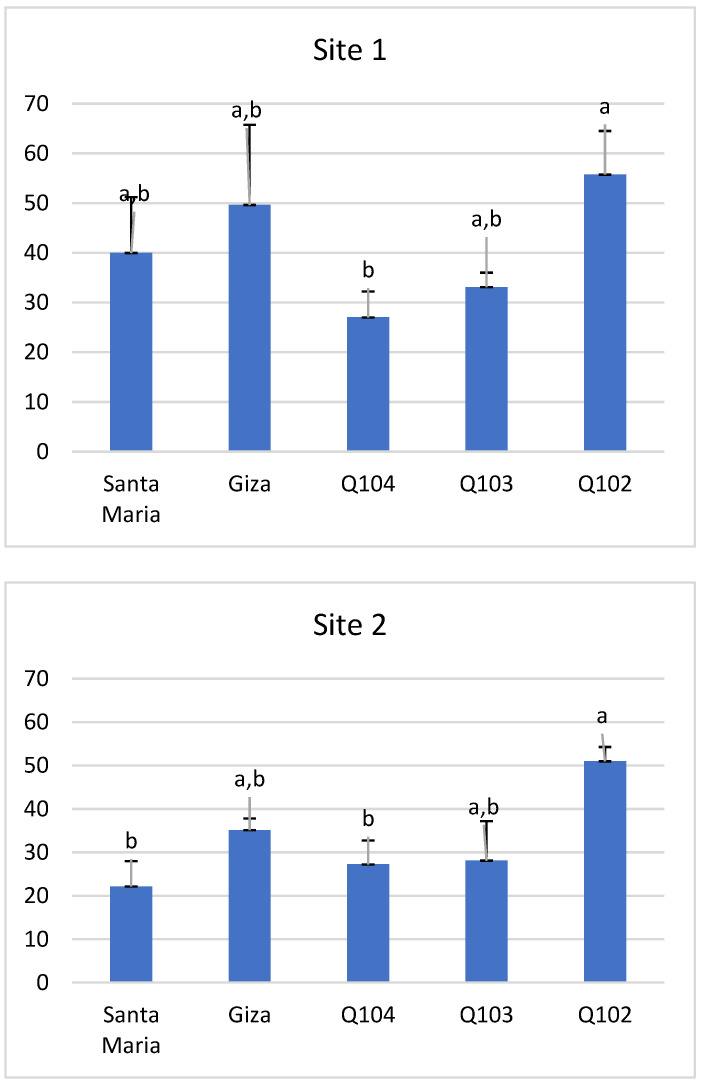
Plant height (cm) at both study sites. *Means with different letters showed statistical difference (p ≤ 0.01 for site 1 and p ≤ 0.02 for site 2)*.

The plant height of the quinoa varieties showed a significant difference in each of the two sites (site 1 *p* < 0.01 and site 2 *p* < 0.02). Genotype Q102 recorded the greatest height, with an average of 55.73 and 50.97 cm in sites 1 and 2, respectively (Figure 6).

The lowest height was noted for genotype Q104, with an average of 27.01 cm in site 1, and for Santa Maria with an average of 22.31 cm in site 2.

We observed that the height of the quinoa plant for all of the varieties studied, with the exception of Q104, was always higher under the saline conditions of site 1 (EC = 9.95 mS/cm) than under the non-saline conditions of site 2 (EC = 0.85 mS/cm).

### 3.3. Stem Diameter

**Figure 7:**
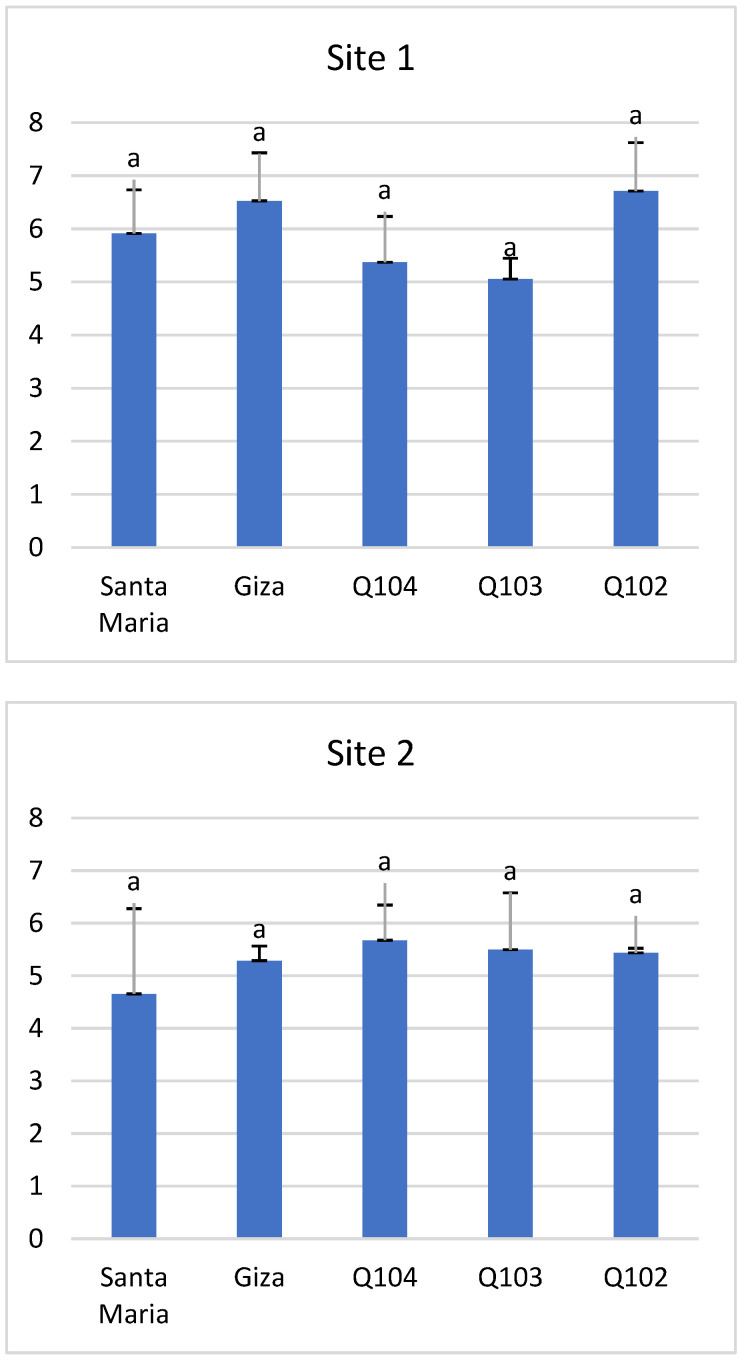
Stem diameter (mm) at both study sites. *Means with different letters showed statistical difference (p ≤ 0.08 for site 1 and p ≤ 0.83 for site 2)*.

For stem diameter, differences between genotypes were not significant at the chosen threshold at each of the two sites (*p* < 0.08 and *p* < 0.83). Locality variation and salinity did not significantly affect stem diameter, which varied between 4.66 mm and 6.71 mm (Figure 7). Genotypes Q102 and Q104 showed the highest diameters on site 1 (6.71 mm) and site 2 (5.67 mm), respectively. This criterion is a possible selection criterion for good stability of large panicle plants, and it gives them a resistance factor when the plants are exposed to wind. In the Saharan region of Ouargla in Algeria, the fields in the desert are very exposed to climatic phenomenons. The wind velocity is one of them and the resistance of quinoa through the diameter of the stems is of importance for crop adaptation.

### 3.4. Grain Yield

**Figure 8:**
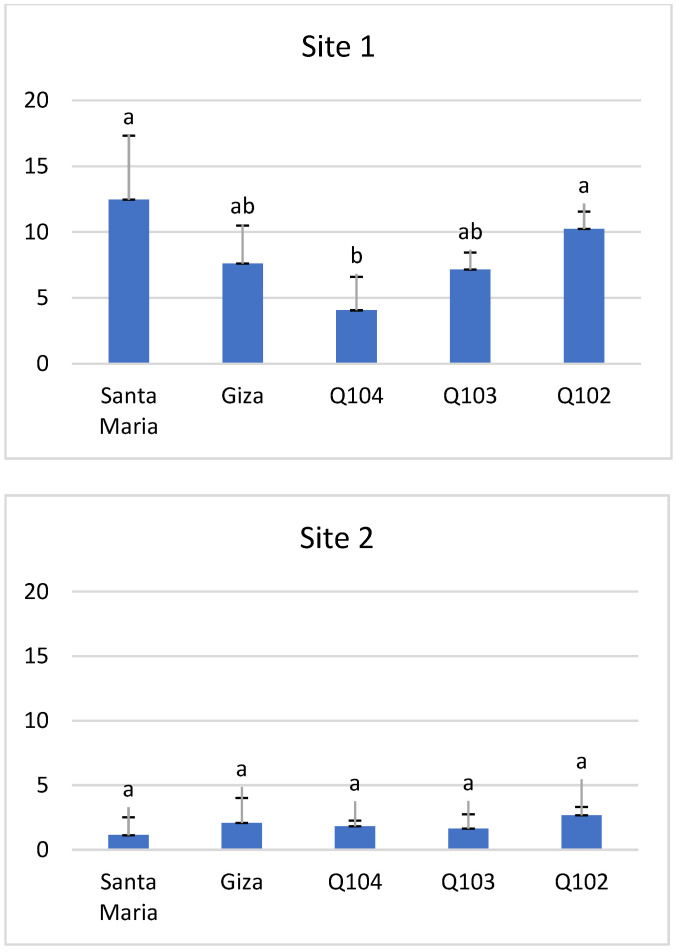
Seed/plant yield (g) at the two study sites. *Means with different letters showed statistical difference (p ≤ 0.01 for site 1 and p ≤ 0.78 for site 2)*.

The study of grain yield presented in Figure 8 showed a significant difference (*p* < 0.01) in site 1. The highest yields are recorded for the Bolivian variety *Santa Maria* (12.47 g/plant), followed by the Peruvian Q102 (10.24 g/plant), and then by the varieties Giza1 and Q103. The variety with the lowest grain yield was Q104 (4.06 g/plant).

In contrast, the statistical differences were not significant (*p* < 0.78) for site 2. *Santa Maria* scored the lowest yield, with an average of 1.13 g/plant, and the highest yield was obtained in the variety Q102, with an average of 2.67 g/plant, and in Giza1 (2.08 g/plant) (Figure 8).

### 3.5. Harvest Index

**Figure 9:**
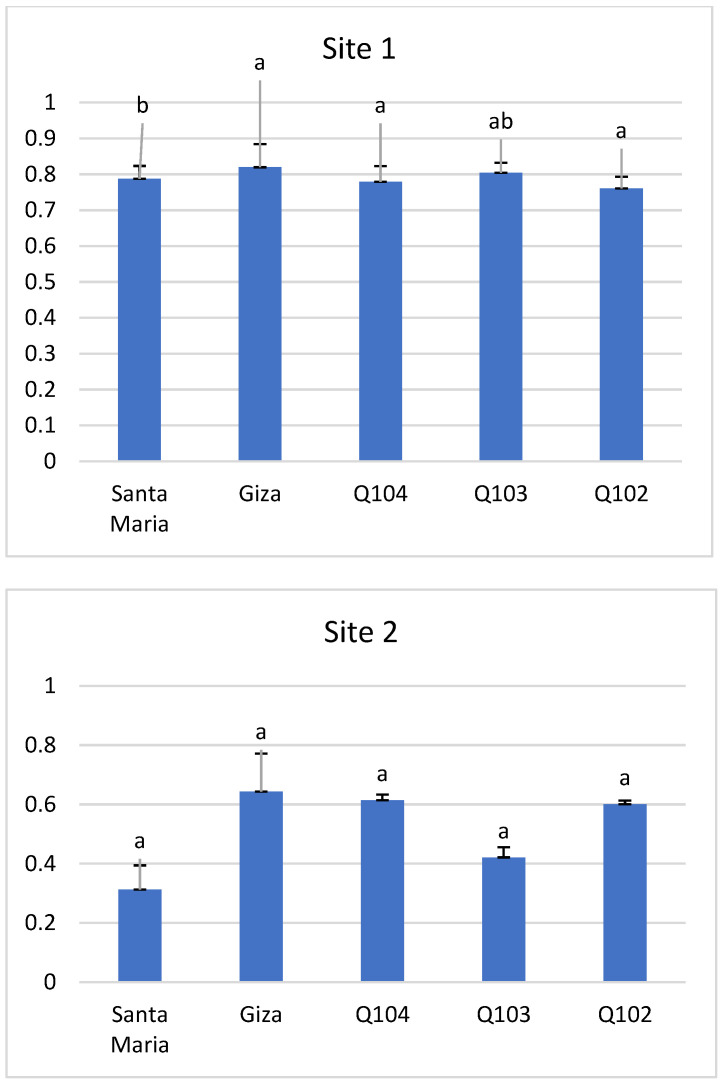
Harvest index at the two study sites. *Means with different letters showed statistical difference (p ≤ 0.43 for site 1 and p ≤ 0.42 for site 2). No significant differences*.

Bhargava et al. [64] and Bertero et al. [65] reported that differences are generally significant in the quinoa harvest index by variety and locality, which is consistent with our results. The harvest index (Figure 9) varied considerably between the two sites. The highest indices were recorded in site 1, where Giza1 and Q103 had the highest indexes (0.82 and 0.80), with non-significant differences (*p* < 0.43) between the genotypes.

The values of the harvest indexes of site 2 were lower compared to those of site 1, varying between 0.64 and 0.31, with the most important again observed at the level of genotypes Giza1 and Q104 (0.64 and 0.61).

## 4. Discussion

The results of this research clearly show the capacity of quinoa to adapt and tolerate the extreme agro-climatic factors of the Ouargla region (southern Algeria), which is characterized by its aridity, drought and soil salinity. The ripening time of all varieties was shorter at site 1 compared to site 2.

The estimate of the total growth duration of the five varieties studied in southern Algeria was between 136 and 164 days. The total growth time of all varieties was short at site 1 (136–162 days) compared to site 2 (152–164 days) and this variation may be due to differences in temperatures, which are always strongly influenced by altitude.

Jacobsen and Stolen [26] reported that the total growth duration in South America was between 110 and 190 days, while in northern Europe the total duration was somewhat shorter (109–182 days) [66]. In northern India, Bhargava et al. [64] reported a total duration of between 109 and 163 days. In the latter cases, it was a spring crop (sowing in November for harvest in February), while in our case, we tested quinoa as a winter crop (sowing in September and harvest in March).

In addition, the differences between the temperature requirements of the varieties justify the contrast between their number of days of ripening. These results are similar to those provided by Szilagyi and Jornsgard [67] and Tan and Temel [68], who reported that quinoa genotypes require different daylight hours and temperatures, while their maturation phases are also different in Romania and Turkey. In site 2, where sowing was late, the duration of growth was also longer compared to site 1, contrary to results reported by Tan and Temel [69], who conducted experiments in the provinces of Erzurum and Iÿdÿr in Eastern Anatolia, where quinoa was then a summer crop (April–September). Quinoa genotypes reached maturity earlier under Erzurum’s conditions, where sowing was late, compared to Iÿdÿr’s conditions, where quinoa matured later when planted earlier.

The highest plant height was that of the late-ripening Peruvian variety Q102 in both study sites. These results corroborate those of Tan and Temel [69], who revealed in their trials in Turkey that late-ripening varieties, such as Oro de Valle and Mint Vanilla, grew higher than those that matured early, such as Q-52 and Moqu Arrochilla. These authors pointed to both genetic differences between varieties and variations in the environment as reasons for their results.

Our results revealed that plant heights differed between varieties within the same site, and this is explained by intrinsic genetic differences. Similar results were found in different geographic regions (Pulvento et al. [70], Bhargava et al. [64], Tan et al. [68]). These authors observed differences in plant heights between varieties in different regions, namely southern Italy in a Mediterranean region with a sub-humid climate (summer crop-sowing in May), in Eastern Turkey (Anatolia province) as a summer crop (sowing in April) and in northern India as a spring crop (sowing mid-November).

With the exception of Q104, the plant height of all of the varieties studied was greater under the saline conditions of site 1 (EC = 9.95 mS/cm) than the non-saline conditions of site 2 (EC = 0.85 mS/cm). This result is not in line with those of Hirich et al. [71] and Hirich et al. [72], according to which the salt tolerance threshold is equal to 9 mS/cm, which normally should lead to increased stress on plants, and, as a consequence, reduced growth. These authors showed in their trials that the increase in salinity (EC = 8 dS/m) negatively affected plant height and led to a severe reduction of 73% compared to EC = 1 dS/m.

The Santa Maria, Q102 and Giza 1 genotypes can be considered to be high-yielding varieties. This reflects a greater adaptability of these quinoa varieties to the agro-climatic conditions of southern Algeria. The differences observed in grain yield between varieties can probably be explained by the intrinsic performance of the varieties and their tolerance to salinity. It was noted that variability was not related to geographical origin. This is illustrated by the Peruvian varieties, of which Q104 had the lowest yields. However, high and medium grain yields were found for varieties Q102 and Q103, which corroborates the results of Bhargava et al. [64] in their trials in India where they found strong significant differences in yield between Bolivian varieties. This variation is very marked when analyzing the harvest index between the two sites, which can be explained by the late sowing in site 2. This led to a proportional increase in temperatures during flowering that was especially detrimental to the late-ripening variety, with the corollary of a decrease in yield and harvest index [73].

## 5. Perspectives

Quinoa is a crop that has attracted attention in recent decades and has been the subject of extensive research recently carried out in all regions of the world. This research has confirmed that quinoa can tolerate various abiotic stresses, including salinity, and that it is an example of an alternative crop in regions that are characterized by a harsh climate, with excessive heat, severe drought and high salinity [71,72,74,75]. Our research is among the first studies in Algeria that can be compared with previous studies conducted on the adaptation of the quinoa species around the world. Due to the increasing problems of salinity in the world, especially in arid areas, and the need for new alternative crops that are more adapted to difficult conditions (saline, drought, high temperature), the results of this research validate the potential of quinoa to be introduced into the cropping systems of the Ouargla area, which are based on phoeniciculture and various associated crops including alfalfa and barley, but also potatoes, fodder corn and cereal cultivation under pivot with durum wheat and soft wheat. Unfortunately, salinity is becoming a major constraint affecting cereal production in the arid zone of Algeria. This constraint is responsible for the drop in yield and is becoming a major evaluation criterion for agricultural development in these regions [76]. Production is approximately 3.6 t/ha [77], the thresholds for a 100% reduction in yields have now been reached, and there will be no more cereal production in 48 to 70 years due to the constant increase in salinity [76].

Nevertheless, after the introduction of quinoa in the Algerian Sahara, the results of this research and of other demonstration trials at research stations in the drylands of Algeria corroborate studies conducted in Morocco and Egypt on the suitability of quinoa for adaptation in drylands. This study indicates that quinoa could be proposed for crop diversification, integrating it into existing cropping systems, as an under-crop in palm groves (fodder crop) and in rotation with field crops (wheat and maize), in order to enrich current cropping systems with alternative species and increase their sustainability in this region.

Like all of the lower Sahara, the Ouargla region is characterized by a desert climate, with large thermal amplitudes between the minima and maxima, and very low rainfall of about 50 mm per year [60]. It has already been pointed out that very high temperatures present an important constraint for the choice of crops [22]. In our case, high (maximum) temperatures, especially in summer, can exceed 50 °C (July), and the average monthly minimum temperature is 4.5 °C in winter (January). The seasonal distribution of low and high temperatures is an essential criterion to properly position the cultivation cycle with an optimal sowing date that avoids the risk of frosts for seedlings and allows plants to develop until flowering before the onset of very high temperatures.

Quinoa is a hardy halophyte plant that can adapt to different geographical areas and abiotic stresses, including drought, frost and heat stress [6,23,71,78]. Quinoa can tolerate a wide range of temperatures (−8 °C to 35 °C) depending on genotypic characteristics and the phenological stage [79]. Despite its adaptation outside its geographical area and its resistance to various abiotic stresses including heat stress, high temperatures during the germination and flowering phase significantly affect the plant and the grain yield in particular. In this context, several studies have focused on the tolerance of quinoa to heat stress (Pulvento et al. [70]; Peterson and Murphy [80]; Yang et al. [81]; Lesjak and Calderini. [82]; Alvar-Beltrán et al. [83]). These studies analyzed the effect of heat stress on different stages of quinoa development including germination and flowering. Lesjak and Calderini [82] in Chile reported a decrease in seed yield when the flowering temperature reached 34 °C. In Italy, it has been shown that the Titicaca variety responded negatively to high temperatures, with a decrease in seed yields to half when the flowering period occurs, around July [70]. Other studies have shown that higher temperatures (20 °C to 25 °C) may promote quinoa growth compared to lower temperatures (8 °C to 18 °C) [81]. In addition, based on tests conducted in Burkina Faso and in the Sahelian, MENA and Mediterranean regions, Alvar-Beltrán et al. [83] found that high temperatures (between 34 °C and 38 °C) on cv. Titicaca causes seed yield losses (25% reduction). For this reason, 38 °C was considered the maximum temperature threshold at flowering. The same authors also found that at temperatures above 34 °C, there was a decrease of over 50% for seed germination.

As quinoa has been introduced in Algeria only recently, farmers’ knowledge and know-how regarding this plant remain very limited. This work therefore proposes a new approach to the optimal period for growing quinoa in the region of Ouargla and Oued Righ to avoid extreme heat. Based on meteorological data (monthly mean temperature-MMT) provided by CRU-TS4.03 [84] downscaled with World Clim2.1 [85], over a period of 28 years (1990–2018) we produced 12 maps to assess the monthly climate risk of high temperatures in the two Saharan regions of Ouargla and Oued Righ using ArcGis10.9 software (ESRI-France: 92195 Meudon, France).

**Figure 10:**
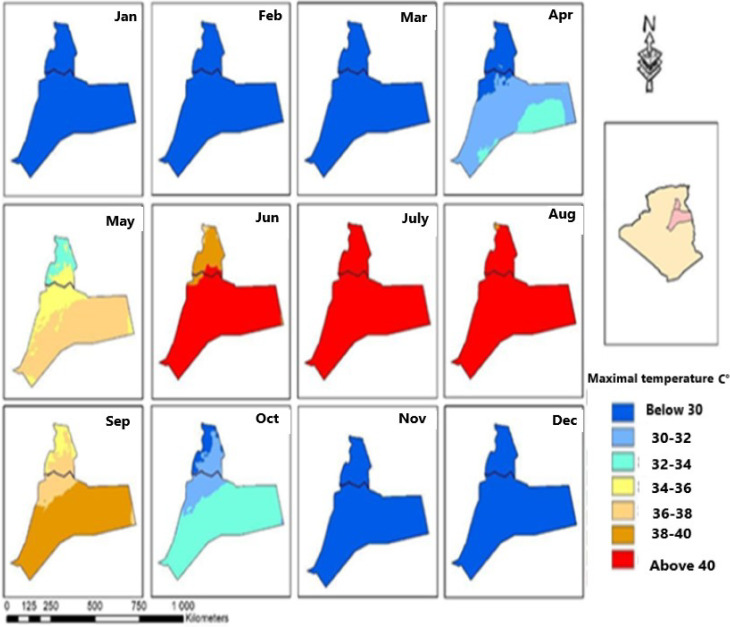
Mean monthly temperatures in two regions in southeastern Algeria (1990–2018).

Based on previous studies, it appeared essential to represent the distribution of the maximum average temperature over the entire year in the study area (Ouargla and Oued Righ region) in order to identify the appropriate period for quinoa cultivation in these regions outside the risk periods (Figure 10).

This figure shows the automatically generated monthly average temperature classes. The first blue class groups MMTs below 34 °C, which are very adequate for the growth of quinoa and are very close to the ideal temperature (optimal between 15 °C and 20 °C) [12,21,66,83,86,87], and correspond to the October-May period for the northern part of the Oued Righ region, and October–April for the Ouargla region. In the second yellow class, the MMTs vary between 34 °C and 38 °C, which are the limit temperatures according to Mamedi et al. [87]. The highest seed germination percentages are between 0 °C and 35 °C, which can regress to 40 °C, thus Alvar-Beltrán et al. [83] reported that most seed yield losses (25% reduction) occurred between temperature levels of 34 °C and 38 °C. For this reason, 38 °C was considered the maximum temperature threshold at flowering, which corresponds to the period of September and May for the region of Ouargla and the south of Oued Righ.

In the brown and red third class, MMTs are above 38 °C, the critical MMT threshold to avoid the coincidence with the period of flowering and germination, and they correspond to the period from June to August for the northern part of the region of Ouargla and Oued Righ, and to September for the southern part.

To avoid high temperatures during the flowering period in the region of Ouargla and Oued Righ in southeast Algeria, we suggest the September–May growing period. It is preferable to sow in September for long-cycle varieties and in October for short-cycle varieties in order for flowering to occur between the months of December–January, when MMTs are adequate (below 30 °C), and also to avoid the frequent, high-speed winds (ranging from 19 to 28 m/s) that occur between March and June.

These high MMTs to be avoided for the cultivation of quinoa give us a first indication for the positioning of the cultivation cycle according to the seasons. However, more detailed work on a daily scale is still necessary to better take into account the real risk incurred by quinoa plants.

## 6. Conclusions

A great variability between genotypes with the same geographical origin was observed, in particular between Peruvian varieties. Q102 showed very high yields and morphological traits at above-average values, whereas values for Q103 were low, as well as for most traits of Q104. This confirms that the variability was not only related to the geographical origin of the varieties but to genetic factors intrinsic to each variety tested.

All of the varieties selected for the trials in this study performed well on the site where saline levels were very high. It can be concluded that the cultivation of quinoa is possible in the environments affected, even strongly, by salinity. The development of the quinoa plant under these conditions manifests acceptable morphological and agronomic traits. This study shows that varieties Q102, Giza1, Santa Maria and Q103 seem to be best adapted to conditions in southern Algeria. The limitations mentioned in the discussion encourage us to extend the evaluation of this crop in other agro-ecological conditions to better assess its adaptation potential in the arid zones of Algeria.

## Figures and Tables

**Table 1:** Table of bioclimatic zones in Algeria.

Bioclimatic Zones	Annual Rainfall (mm)	Percentage of Total Area (%)
Humid	900–1800	0.4
Sub-humid	600–900	1.42
Semi-arid	300–600	4.12
Arid	300–100	4.78
Saharan	<100	89.5

*Own elaboration adapted from the Commissariat for the development of agriculture in the Saharan regions (CDARS/Ministry of Agriculture and Rural Development, 2017)* [54].

**Table 2:** Climate data for the study area (October–April) during the crop cycle of the 2019/2020 crop year.

Month	Max Temperature (°C)	Min Temperature (°C)	Relative Humidity (%)
October 2019	31	17.2	35.8
November 2019	23.3	9.3	37.3
December 2019	21.1	7.1	46.1
January 2020	19	3.2	46.1
February 2020	23.4	6.7	35.5
March 2020	25.8	11.3	33.3
April 2020	30.7	16.4	29

Data source: National Meteorological Office (O.N.M) Ouargla.

**Table 3:** Characteristics of quinoa genotypes used.

Genotypes	Origin	Institution	Seed Color
Santa Maria	Cultivar of Bolivia	ITDAS	White and brown
Giza1	Cultivar of Egypt	ITDAS	Beige
Amarilla Sacaca (Q102)	Variety from Peru	ITDAS	Orange
Kancolla (Q104)	Variety from Peru	ITDAS	Yellow, brown and beige
Q103	Variety from Peru	ITDAS	Yellow, brown and beige

## Data Availability

Data available only on demand and after the PhD thesis presentation (K.M.).
